# Improved Power Conversion Efficiency with Tunable Electronic Structures of the Cation-Engineered [A_i_]PbI_3_ Perovskites for Solar Cells: First-Principles Calculations

**DOI:** 10.3390/ijms232113556

**Published:** 2022-11-04

**Authors:** Ahmed Al-Shami, Anass Sibari, Abdallah El Kenz, Abdelilah Benyoussef, Amine El Moutaouakil, Omar Mounkachi

**Affiliations:** 1 Laboratory of Condensed Matter and Interdisciplinary Sciences, Physics Department, Faculty of Sciences, Mohammed V University in Rabat, Rabat BP 1014, Morocco; 2 Modeling, Simulation & Data Analysis Program, Mohammed VI Polytechnic University, Lot 660, Hay Moulay Rachid, Ben Guerir BP 43150, Morocco; 3 Department of Physics, Faculty of Science, Sana’a University, Sana’a 12544, Yemen; 4 Supramolecular Nanomaterials Group, Mohammed VI Polytechnic University, Lot 660, Hay Moulay Rachid, Ben Guerir BP 43150, Morocco; 5 Hassan II Academy of Science and Technology, Mohammed V University in Rabat, Rabat BP 1014, Morocco; 6 Electrical and Communication Engineering, College of Engineering, UAE University, Al Ain P.O. Box 15551, United Arab Emirates; 7 Institute of Applied Physics, Mohammed VI Polytechnic University, Lot 660, Hay Moulay Rachid, Ben Guerir BP 43150, Morocco

**Keywords:** power conversion efficiency, perovskites, photovoltaic, solar

## Abstract

Higher power conversion efficiencies for photovoltaic devices can be achieved through simple and low production cost processing of ${\mathrm{APbI}}_{3}\left(A={\mathrm{CH}}_{3}{\mathrm{NH}}_{3},{\mathrm{CHN}}_{2}H_{4},\ldots \right)$ perovskites. Due to their limited long-term stability, however, there is an urgent need to find alternative structural combinations for this family of materials. In this study, we propose to investigate the prospects of cation-substitution within the A-site of the ${\mathrm{APbI}}_{3}$ perovskite by selecting nine substituting organic and inorganic cations to enhance the stability of the material. The tolerance and the octahedral factors are calculated and reported as two of the most critical geometrical features, in order to assess which perovskite compounds can be experimentally designed. Our results showed an improvement in the thermal stability of the organic cation substitutions in contrast to the inorganic cations, with an increase in the power conversion efficiency of the Hydroxyl-ammonium (NH_3_OH) substitute to η = 25.84%.

## 1. Introduction

Perovskite materials with the ABX_3_ (A = cations, B = Pb, and X = I) formula have a variety of valuable properties, including both in application-oriented uses and fundamental physics [1,2,3]. One of the main reasons perovskites are so successful is their adaptability—they can be tailored to have a variety of different properties, which allows them to meet specific requirements such as an impressive series of improvements in light to electricity efficiency [4,5,6,7,8]. In just over five years of exhaustive research, the efficiency of (CH_3_NH_3_)PbI_3_ has been around 25% [9,10]. In this family, the perovskite architecture is largely retained, with one ion substituted by an organic ion (usually A or X). This makes the perovskite a hybrid with A and B as the cations, and X as a halogen or oxygen ion—two of the most common anions—while different metal cations can form halide and oxide perovskites [11,12] where the radii of A is larger than that of B. Both these perovskites have crystal structures that are very similar, with BX_6_ octahedra located in between the A atoms [13,14]. Studies from first-principles calculations showed that adding substituents such as Br, Cl and F to MAPbI_3_ can help facilitate the transfer of charge from the hybrid perovskite to the electrodes [2]. On the other hand, B-site substitutions change the bandgap. In our study, we investigated how the cation substitution at the A-site might affect the organic perovskite’s properties. Our analysis suggests that substituting the A cation may be a way to obtain an optimal band gap (1.1–1.4 eV) and high power conversion efficiency [9,15]. The Goldschmidt tolerance factor (*t*) has been a key element in the development of perovskites for many years, and it has been expanded to the growing field of organic-inorganic perovskites [4,16,17,18]. The *t* value should range between 0.8 and 1.11—otherwise, the crystal structure will be distorted, and might be destroyed. A smaller *t* value will result in a tetragonal or orthotopic shape with low symmetry. The *t*-value of an ideal cubic structure is from 0.89 to 1.0. The ideal value of the octahedral factor (*μ*), on the other hand (044 to 0.90), affects the stability of the octahedron, and further influences the stability of the perovskite structure [19,20].

**Figure 1:**
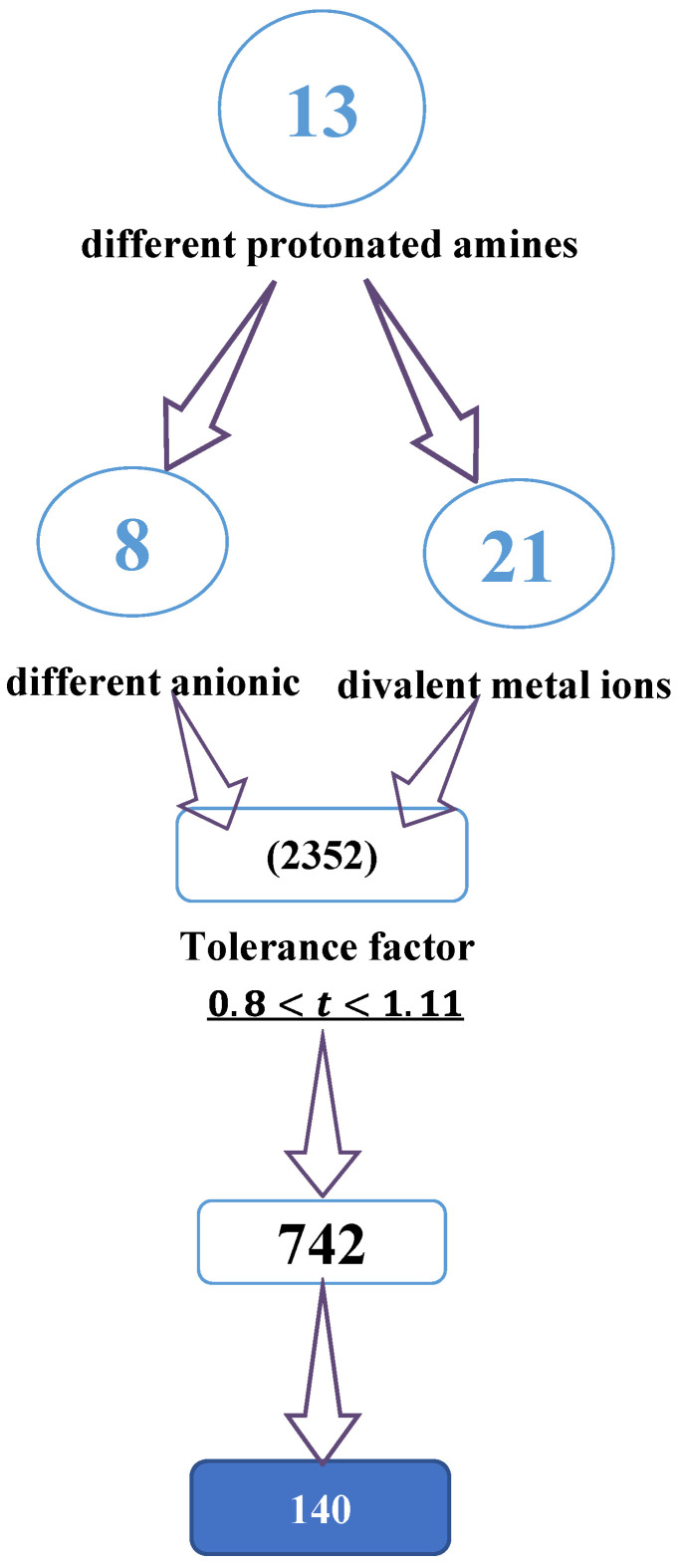
Diagram showing the selection process for cations using the tolerance factor.

As shown in Figure 1, recent machine learning studies generated about 2352 cations from different protonated amines. Only 742 of these cations showed a tolerance factor (*t*) of between 0.8 and 1.11, from which about 140 materials are better identified, such as well-characterized hybrid perovskites [13,21].

**Figure 2:**
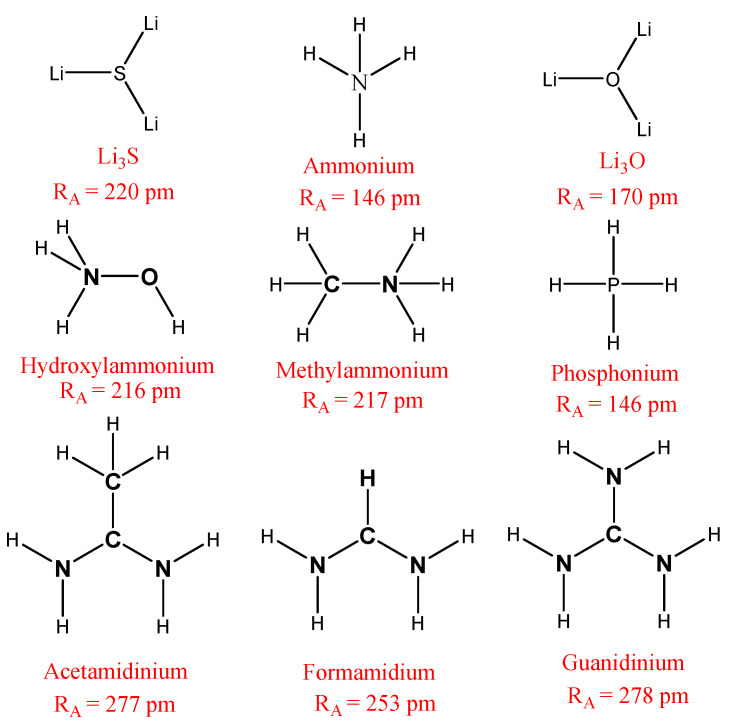
The chemical formula for the cations studied in this paper.

In our paper, we limited our study to nine cations (CH_4_, PH_4_, Li_3_O, CH_3_OH, Li_3_S, CH_3_NH_3_, CH(NH_2_)_2_, CH_3_C(NH_2_)_2_ and C(NH_2_)_3_), whose chemical formulas are detailed in Figure 2, and which have ionic radii of 146, 167, 170, 216, 220, 222, 253, 277 and 278 pm, respectively [22,23].

For all substitutions (except for Li_3_O and Li_3_S), the short circuit current (*J*_SC_), the open-circuit voltage (*V*_OC_) and the power conversion efficiency (PCE) are also calculated. We hypothesized that every photon energy above *E* is absorbed, always promoting the perovskite to generate electron-hole pairs [20,24,25,26,27,28,29,30,31].

*V*_OC_ was calculated, as well as the maximum theoretical limit of the PCE for all configurations, where the efficiency is regarded as a function of the band gap energy (*E*_g_) and the total incident power density (*P*_S_), which can be calculated using solar spectrum data. PCE was obtained by using *J*sc, *V*oc, and the fill factor (FF), as well as *P*_S_ [32,33,34,35].

## 2. Results and Discussion

We used density functional theory (DFT) and ab-initio molecular dynamics (AIMD) to investigate the crystal structure, electronic structure, thermal and dynamic stability, and the power conversion efficiency of the substituted cubic MAPbI_3_ (MA = CH_3_NH_3_^+^).

### 2.1. Optimization

**Figure 3:**
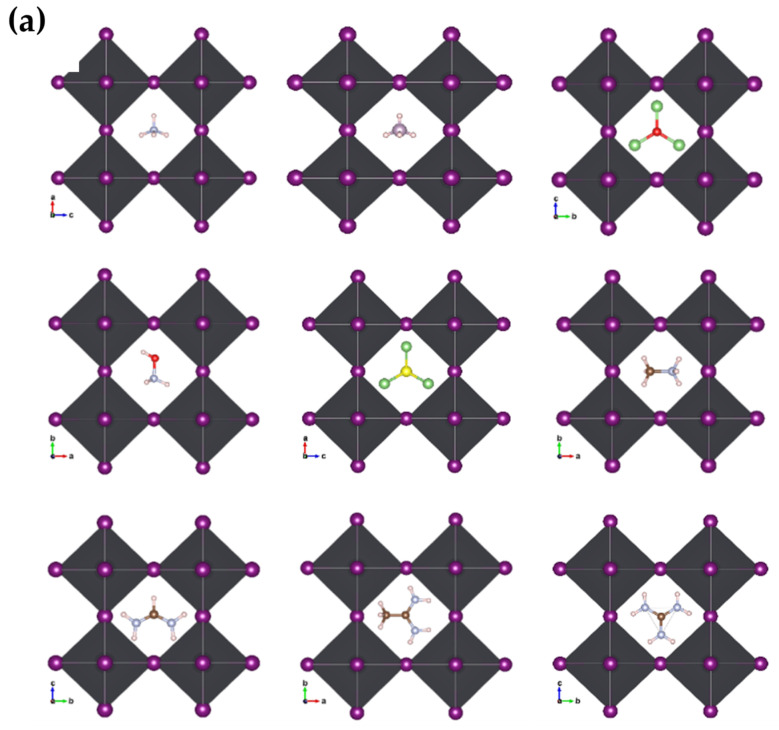

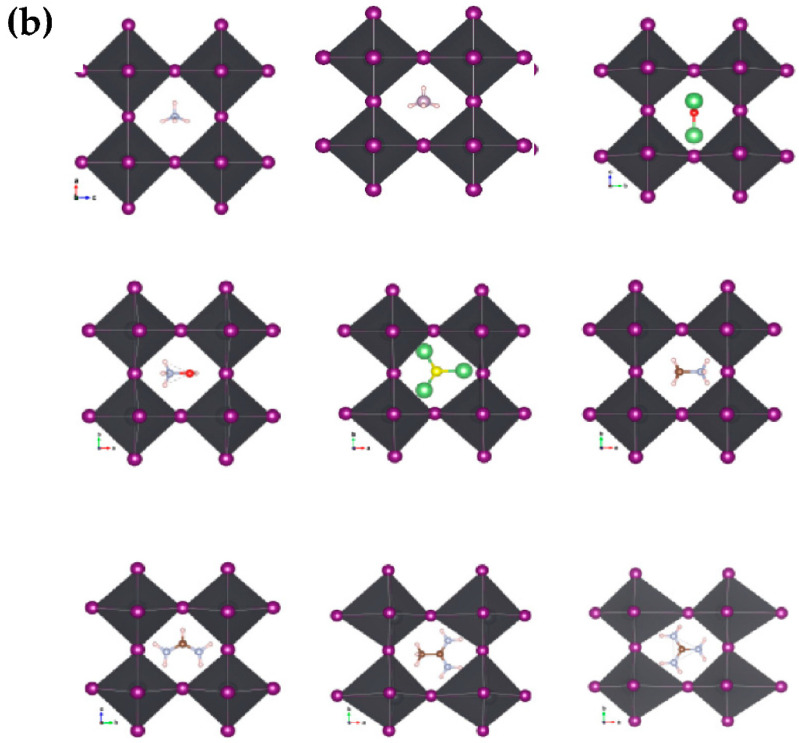
Geometric structures of A_i_PbI_3_ with different cation: (**a**) before optimization; and (**b**) after optimization with Van der Waals correlation.

We optimized the structures after substituting the cations while observing the distortions in the nascent crystallites relative to the pristine crystal of MAPbI_3_. We noticed clear distortions in some structures, especially those with larger cations, or inorganic (Li_3_S, Li_3_O) size cations, while the other structures did not change significantly. Figure 3a,b shows the engineered structures before and after optimization for all the studied systems. Table 1 gives an exhaustive list of all calculated parameters [14,36]. This is attributed to many reasons, such as the difference in electrical polarization between the anion and the cation, as well as the size of the cation in relation to a unit cell.

### 2.2. Tolerance Factor and Octahedral Factor and Parameters Optimization

In our study, we substituted the MA cation with a number of different-sized organic cations and their constituting atoms within the cubic phase, and for a first examination of the stability of the crystal lattice with each cation, we calculated the tolerance factor (*t*) and the octahedral stability factor (*μ*), and matched them with the permissible ranges, which were arranged in an ascending order of size, as shown in Table 2. The results indicated that every one of these falls inside the stable range [37].

### 2.3. Stability

Formation Energy:

We can clearly see that this perovskite can be produced in a laboratory based on the calculated formation energy values. Additionally, we also noticed that the formation energy rises as the cation size increases in all structures (see Table 1). The reason may be attributed to the poor thermal stability. The formation energies were calculated by Equation (1)

(1) $$E_{F}^{\mathrm{Ai}}=\frac{E_{\mathrm{Total}}^{A_{i}{\mathrm{BX}}_{3}}-{\mathrm{yE}}_{\mathrm{Total}}^{{\mathrm{BX}}_{3}}-{\mathrm{xE}}_{\mathrm{Total}}^{A_{i}}}{x+y}\;$$

where A_i_ is the different cation, and x and y are the numbers of A_i_ and BX_3_ atoms in a unit cell, respectively.

### 2.4. Dynamical Stability

**Figure 4:**
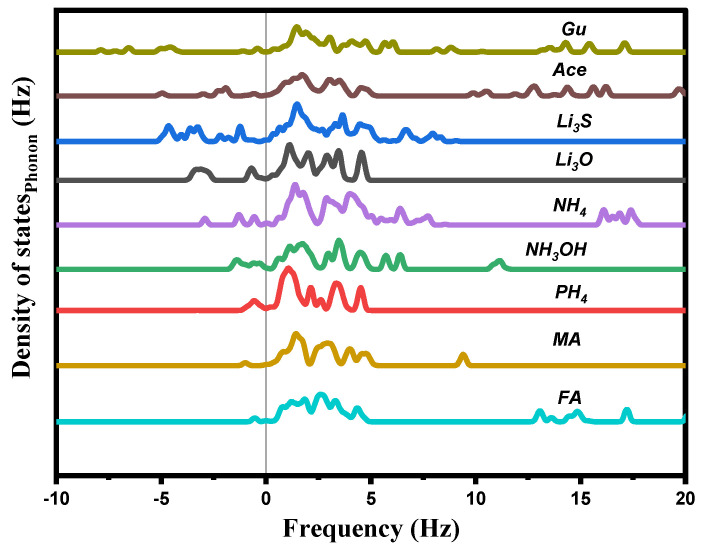
Calculated total phonon density of states Ph−DOS for A_i_PbI_3_ with the different substituting cations.

The total phonon density of states (Ph-DOS) is calculated at the equilibrium volumes for different cations of A_i_, and displayed in Figure 4. For all these cations, a small imaginary frequency was observed for the CH_4_, PH_4_, CH_3_OH, CH_3_NH_3_, CH(NH_2_)_2_, CH_3_C(NH_2_)_2_, and C(NH_2_)_3_, indicating that these structures are stable or at least relatively stable at ambient conditions. Other than this, a clear imaginary frequency was observed in the alkali (Li_3_O, and Li_3_S) structures, indicating that these structures are unstable at ambient conditions.

### 2.5. Thermal Stability

**Figure 5:**
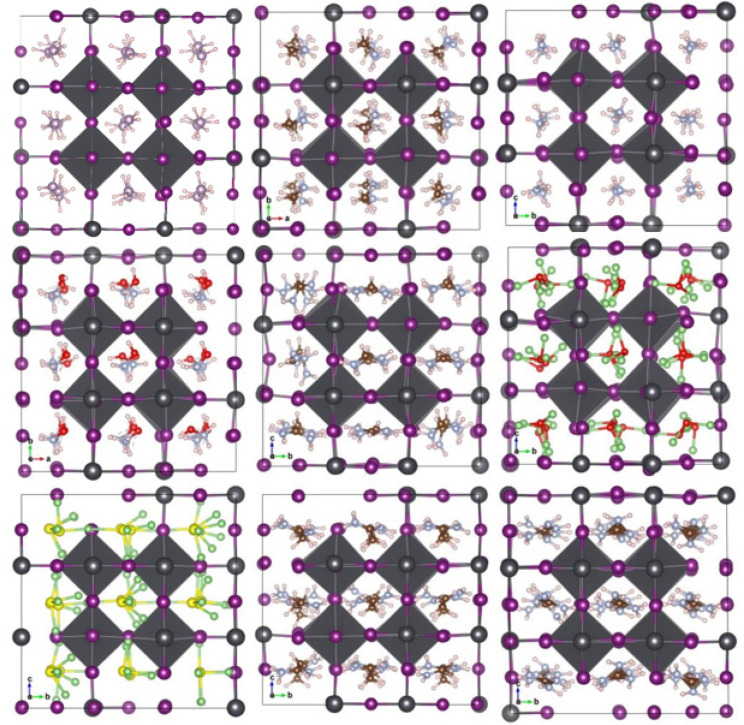
The cubic APbI_3_ perovskites’ structure changed with a 3 × 3 × 3 supercell after ab initio molecular dynamics simulation of 50 ps at 300 K. Atomic colors are: Pb (grey); C (brown); H (white); F (blue); Li (cyan); Cl (green); N (silver); O (red); S (yellow); P (lavender); and I (purple).

**Figure 6:**
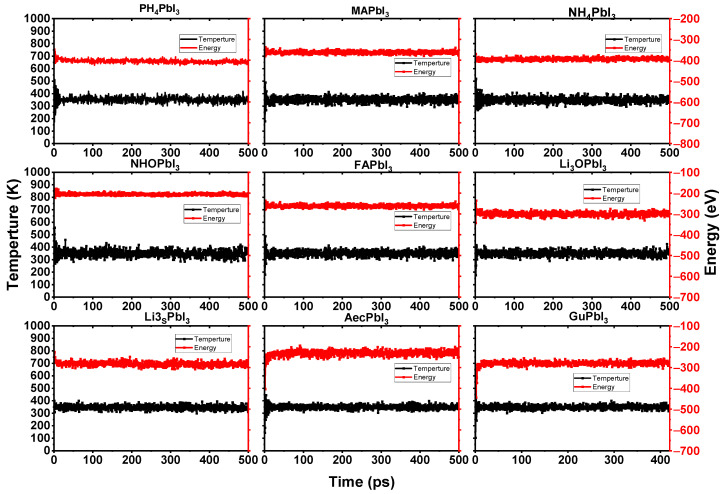
Fluctuation of the temperature and total energy of the considered perovskite systems from 0 to 500 ps using AIMD simulations at 300 K.

Furthermore, we simulated the chemical reaction using an ab-initio molecular dynamics simulation that was based on the force calculations from density functional theory. The simulations were run on models with 3 × 3 × 3 supercells, for a total of 25 ps per case (see Figure 5). The smaller the organic component of a system, the more it is over-correlated with other parts of the system. This suggests that the smaller system is more easily moved around by electrostatic forces. A large-scale system mitigates this finite-size artifact, and produces a more reliable explanation of the anisotropic rotational behavior of cations. To compare the dynamics performance of the perovskites with the cations, the AIMD simulations are performed for the cubic NH_4_PbI_3_, PH_4_PbI_3_, Li_3_OPbI_3_, NH_3_OPbI_3_, Li_3_SPbI_3_, MAPbI_3_, AMPbI_3_, AcPbI_3_ and GuPbI_3_ perovskites with a 3 × 3 × 3 supercell. Figure 6 shows the AIMD simulation results for cubic Li_3_OPbI_3_ and Li_3_SPbI_3_ perovskites after 25 ps.

According to the AIMD simulation of the A_i_PbI_3_ perovskite depicted in Figure 6, the A_i_PbI_3_ perovskites exhibit no evident structural change after a 500 ps AIMD simulation at 300 K.

The A_i_^+^ ions in the A_i_PbI_3_ perovskites do not congregate, the PbI_3_ frame does not crumble (see Figure 5), and the temperature and energy curves indicate balance with a little shock (see Figure 6).

Furthermore, given the strong interaction between Li_3_O^+^ and Li_3_S^+^ ions in super-alkali perovskites, as well as the large-size cations, investigating the dynamics performance of perovskites based on various cations (A_i_) is critical for identifying perovskites with stable dynamics performance [38]. This may be due to the hydrogen bonding between the cation and anions (PbI_3_ frame) and the difficulty of movement of the cation inside the cavity of the structure, due to its large size, and might also be attributed to the electrostatic forces resulting from the transfer of charges between positive cations A+ and negative anions PbI6- (see Figure S3).

### 2.6. Electronic

**Figure 7:**
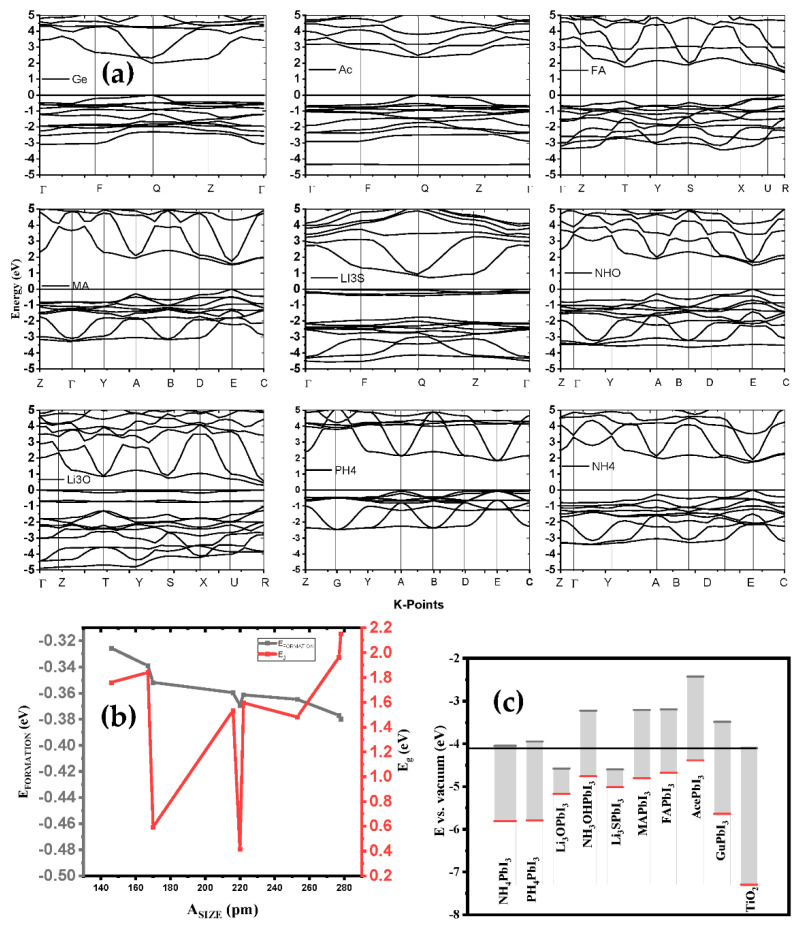
(**a**) The variation of formation energy and bandgap function of the different cations; (**b**) the potential energy with A_i_PbI_3_; and (**c**) band structure of A_i_PbI_3_ in comparison with Van der Waals.

In photovoltaic applications, determining the absorber layer material with appropriate electronic features plays an important role in improving device performance. The size of a band gap is important in estimating the number of photons that are absorbed, and the rate of photogenerated charge carriers with minimal optical losses. To generate and transport free charge carriers efficiently, low exciton binding energy and low effective masses of holes and electrons are necessary. Moreover, DFT studies reveal that, apart from LiO_3_, LiS_3_ and Gu, the electrical structures of the examined substituted perovskites were like those of MAPbI_3_, although their bandgaps marginally decreased or increased compared to those of MAPbI_3_. Our findings imply that adjusting the band gap and enhancing thermal and dynamical stability may both be accomplished by substitution in halide perovskite. To help us better understand the electronic structure of the optimized A_i_PbI_3_ perovskite structures, we calculated their band structures using the GGA-PBE method for A_i_ = NH_4_, PH_4_, Li_3_O, NH_3_O, Li_3_S, CH_3_NH_3_, CH(NH_2_)_2_, Ace, and Gu along the high symmetry points of the Brillouin zone, which has taken three space groups—cubic, triclinic and orthorhombic—as shown in Figure 7a. Here, the Fermi level is set to zero. The maximum energy of valence band (VBM) and the minimum energy of conduction band (CBM) for all computed band structures are clearly shown in the figure, to be located along points R, Q and E of the Brillouin zone for (PH_4_, Li_3_O, CH(NH_2_)_2_), (Li_3_S, Ace, Gu-NH_4_) and (NH_3_O, MA), respectively. Since these band gaps represent the direct nature of the band gaps, these materials can be called as having good optical absorption properties (Figure 7b,c). The calculated band gaps for NH_4_PbI_3_, PH_4_PbI_3_, Li_3_OPbI_3_, NH_3_OPbI_3_, Li_3_SPbI_3_, CH_3_NH_3_PbI_3_, CH(NH_2_)_2_PbI_3_, AcPbI_3_ and GuPbI_3_ are 1.7611, 1.6101, 0.5932, 1.5348, 0.4146, 1.5971, 1.4815, 1.9645 and 2.1517 eV, as given in Table 3 respectively, which are in fair agreement with the experimental results and other theoretical calculations.

### 2.7. Optic

**Figure 8:**
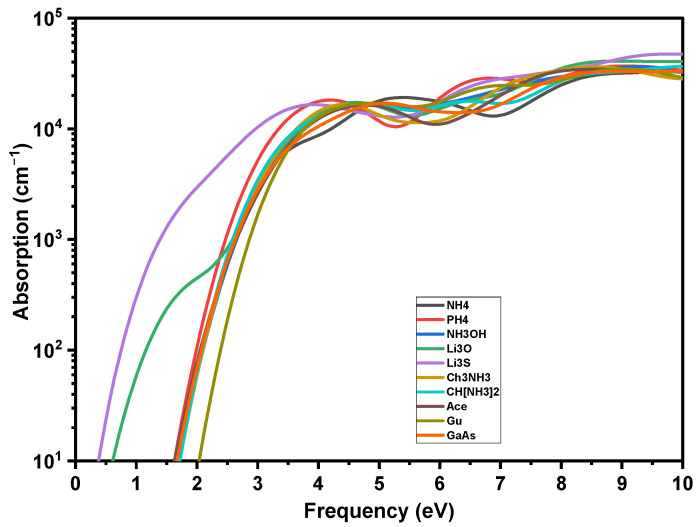
Absorption coefficients of crystalline GaAs and A_i_PbI_3_ perovskite.

The optical properties of the solar cell, including the absorption edge, the intensity of the absorption at various wavelengths and the reflection coefficient, are crucial to determine its external and internal quantum efficiencies (see Equation (S5) in the Supplementary Materials). These optical properties are the result of light waves interacting with its valence electrons. The optical response of a material when it is interacted with an electromagnetic radiation, such as light waves, is given by the complex dielectric functions ε_1_(ω) and ε_2_(ω). For energies greater than *E*_g_, our predicted absorption (*α*) compares favorably to a recent absorption measurement, providing important validation of our technique. According to the experiment (see Figure 8), the calculated *α* is 10^4^ cm^−1^ at the start, and increases linearly (on a logarithmic scale) with energy. The visible slope variation around *α* = 2.5–3 eV replicates a comparable characteristic in the experimental spectrum [39]. The absorption curves of A_i_PbI_3_ and GaAs are identical throughout a large energy range, except for the energy onset corresponding to the different *E*_g_ of the studied materials.

### 2.8. Power Conversion Efficiency

Despite the cation substitution in the identical structures, these power conversion efficiencies were observed to be substantially closer to those of single-junction solar cells (shown in Table 3), which were estimated using Equation (S5) [40]. For each simulation, we considered a power input of *P*_S_ = 100 mW/cm^2^, which is the power under the maximum AM1.5 solar illumination spectrum with *T*_Cell_ = 300 k and *T*_S_ = 6000 k.

**Figure 9:**
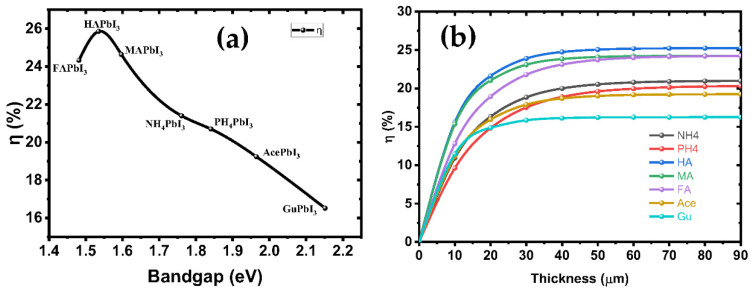
(**a**) The variation theoretical efficiencies under AM1.5 illumination in dependence of the bandgap energy according to the substitution of cation in A-site; and (**b**) the variation in efficiencies function in thickness of all structures.

The electron-hole pairs are easily separated and moved in the CBM and VBM states, which suggests that the perovskite-based solar cells studied so far have the potential to have high PCE, which may give them an edge over other cells in terms of power output. Figure 9a shows the PCE of solar cells based on these perovskites as a function of the band gaps. The cubic HAPbI_3_ perovskite-based solar cells with a PCE of 25.84% are the best option, followed by the MAPbI_3_ perovskites with a PCE of 24.63%. Experimental reports have shown that the efficiency of solar cells tends to be around 25.2% [10], while the maximum efficiency conversion is observed for a 1.4–1.6 eV bandgap. Moreover, the cubic GuPbI_3_ perovskite with a direct maximum band gap of 2.15 eV also shows a PCE of 16.52%. The PCE results of these perovskites indicate that they are a good choice for single-junction solar cells. We have calculated PCE for our intrinsic perovskite solar cell with *E*_g_ of perovskite structures shown in Table 3. The efficiency of these structures, according to the Shockley–Queisser model, is represented in Figure 9b. Based on these results, the considered perovskites can reach an efficiency limit of 25.84% in ideal conditions, with *T*_C_ = 300 k and *T*_S_ = 6000 k for the AM1.5 spectrum.

**Figure 10:**
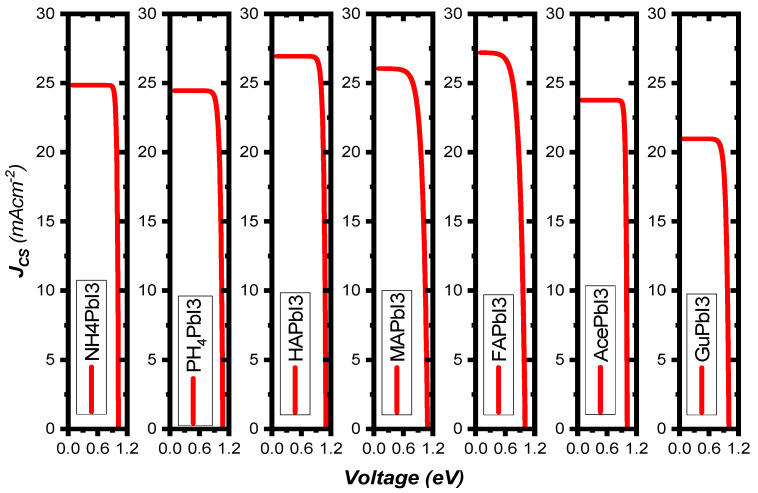
The curves of short-circuit current function open-circuit voltage of A_i_PbI_3_ with different cations under AM 1.5 G illumination at 100 mW/cm^2^.

Figure 9b shows the variation of the PV parameters as a function of the absorber’s thickness. Our results depicted from the simulations show that at a thickness of 60 nm, the efficiency rose as high as 24.63%, while *FF* = 68.28%, *J*_SC_ = 26.04 mA/cm^2^ and *V*_OC_ = 1.09 V. The defects were considered in the simulated absorber layer. We found that the efficiency increases up to 25.84% as the thickness increases from 10 nm to 90 nm, thus representing the increased electron-hole pair generation rate in the absorber MAPbI_3_ [41], while at higher thickness, the recombination of charges starts to occur earlier in the metal, before they reach the contact points—this results in a saturation of charges at thicker metal layers [42]. It was found that the amount of VOC decreased as the thickness of the absorber material, MAPbI_3_, was increased. This was related to the short-circuit current (*J*_SC_) and dark saturation current (*J*_0_), and at higher thickness the dark saturation current rises, which causes the recombination probability of the charge carriers to increase. The Shockley-Queisser Equation (S10) clearly describes the relationship between *V*_OC_, *J*_SC_ and *J*_0_ [43]. Figure 10 illustrates the J-V curves of all structures, and the structure parameters are shown in Table 3.

## 3. Conclusions

In conclusion, we found that there is a high probability of the formation of 3D perovskite structures by calculating tolerance factors and octahedral factors for 486 permutations of ABX_3_ organic-inorganic hybrid compounds reported in the literature. Nine different organic monoammonium cations were studied in combination with Pb metal ions and I halide. We used CH_3_NH_3_^+^ as a reference to estimate the steric bulk of molecular cations. We have found that the structures are dynamically stable and thermally efficient, and as a consequence, the adjustment in A+ revealed a significantly increased PCE of 25.84% in Hydroxylammonium (NH_3_OH), compared to 24.63% in the MA of the control device, as well as an obvious improvement in its operational and thermal stabilities. There are many previously unknown compounds that could form stable perovskite phases at ambient conditions. This study is expected to lead to increased research efforts in designing perovskite structures with specific optoelectronic properties, as well as their great structural flexibility. This, combined with their promising physical properties, makes perovskites a powerful chemical structure for ferroelectrics and future photovoltaics technologies.

## Figures and Tables

**Table 1:** The theoretical lattice constants a, b, c, α, β, γ, and space groups of A_i_PbI_3_ with the different cations.

	a (Å)	b (Å)	c (Å)	α (°)	β (°)	γ (°)	Space Group
NH_4_PbI_3_	6.3079	6.2407	6.3912	90.0000	90.0000	90.0000	P m
PH_4_PbI_3_	6.5455	6.5483	6.5574	90.0000	88.7342	90.0000	P m
Lis_3_OPbI_3_	6.8934	6.9410	6.9572	90.0000	90.0000	90.0000	P m m 2
HAPbI_3_	6.3388	6.3572	6.3517	90.0000	84.496	90.0000	P m
Li_3_SPbI_3_	6.6913	6.6140	6.5984	89.0179	90.4295	90.7352	P1
MAPbI_3_	6.4271	6.4006	6.4547	90.0000	90.1803	90.0000	P m
FAPbI_3_	6.4918	6.5457	6.3295	90.0000	90.0000	90.0000	P m m 2
AcePbI_3_	7.6505	7.0395	6.2866	90.2916	90.0465	90.2715	P1
GuPbI_3_	7.2249	6.5031	6.4847	90.5220	89.8453	89.5245	P1

**Table 2:** Estimated radii (*R*_A_), band gap (*E*_g_), formation energy (*E*_f_), polyhedral factor (*μ*) and tolerance factor (*t*) of (A_i_)PbI_3_ Perovskites with GGA-PBE (vdW) by theoretical calculations.

	*R*_A_ (pm)	*E*_g_ (eV)	*E*_f_ (eV)	*μ*	*t*
NH_4_PbI_3_	146	1.7611	−0.32564	0.468182	0.801242
PH_4_PbI_3_	167	1.8411	−0.34885	0.468182	0.847215
[Li_3_O] PbI_3_	170	0.5932	−0.34170	0.468182	0.853782
HAPbI_3_	216	1.5348	−0.35955	0.468182	0.954485
[Li_3_S] PbI_3_	220	0.4146	−0.34944	0.468182	0.963241
MAPbI_3_	222	1.5971	−0.36116	0.468182	0.967620
FAPbI_3_	253	1.4815	−0.36469	0.468182	1.035485
AcePbI_3_	277	1.9645	−0.37708	0.468182	1.088025
GuPbI_3_	278	2.1517	−0.37980	0.468182	1.090214

**Table 3:** Calculated open-circuit voltages (*V*_OC_), short circuit currents (*J*_SC_) and power conversion efficiencies (*η*) for the hypothetical perovskites A_i_PbI_3_ and MAPbI_3_.

	*J*_CS_	*FF*	*V*_OC_	*η* (%)	*E*_g_
NH_4_PbI_3_	24.84	66.4391	1.02	21.39543	1.7611
PH_4_PbI_3_	24.45	62.3652	1.05	20.34961	1.8410
HAPbI_3_	26.94	68.6404	1.10	25.84328	1.5348
MAPbI_3_	26.04	68.2843	1.09	24.63394	1.5971
FAPbI_3_	27.38	69.4876	1.006	24.32676	1.4815
AcePbI_3_	23.75	63.1115	1.01	19.24151	1.9645
GuPbI_3_	20.97	61.6615	1.005	16.51673	2.1517

## Data Availability

Used data and equations are available in the Supplementary Materials.

## Supplementary Materials

The following supporting information can be downloaded at: https://www.mdpi.com/article/10.3390/ijms232113556/s1, Figure S1: The process of merging a cation A+ into the PbI6- framework to produce perovskite structures; Figure S2: Ionic packing in an ideal cubic perovskite structure, with (a) a loosely packed crystal structure with a small A radius with t < 1, (b) an ideal cubic perovskite structure with 0.8 < t < 1.11 and (c) a tightly packed crystal structure with a large A radius with t > 1; Figure S3 Difference in charge between the A+ cation and the PbI6- anion, where the yellow area represents charge accumulation, whereas the blue area represents charge depletion. References [44,45,46,47,48,49,50,51,52] are cited in Supplementary Materials.
