# Supplementary material for: Improved Power Conversion Efficiency with Tunable Electronic Structures of the Cation-Engineered [Ai]PbI3 Perovskites for Solar Cells: First-Principles Calculations
Source: Int J Mol Sci. 2022 Nov 4;23(21):13556. doi: 10.3390/ijms232113556 (PMC9656733; doi:10.3390/ijms232113556)
Supplement: Supplementary file 1 [file ijms-23-13556-s001.zip › ijms-1985881-supplementary.pdf]

Supplementary information:

Improved power conversion efficiency with tunable electronic structures of the cation-engineered $[A_i]PbI_3$ perovskites for solar cells: first-principles calculations

Ahmed Al-Shami[1,2,3], Anass Sibari[4], Abdallah El Kenz[1], Abdelilah Benyoussef[5], Amine El Moutaouakil[6*] and Omar Mounkachi[1,2*]

1 Laboratory of Condensed Matter and Interdisciplinary Sciences, Physics Department, Faculty of Sciences, Mohammed V University in Rabat, Morocco.
2 MSDA, Mohammed VI Polytechnic University, Lot 660, Hay Moulay Rachid Ben Guerir, 43150, Morocco
3 Department of Physics, Faculty of Science, Sana'a University, Sana'a, Yemen.
4 SNG, Mohammed VI Polytechnic University, Lot 660, Hay Moulay Rachid Ben Guerir, 43150, Morocco
5 Hassan II Academy of Science and Technology in Rabat, Morocco
6 Electrical and Communication Engineering, College of Engineering, UAE University, P.O. Box No. 15551, Al Ain, UAE
* a.elmoutaouakil@uaeu.ac.ae, omar.mounkachi@fsr.um5.ac.ma.

Computational Details:

The results presented in this work were performed using density functional theory (DFT) and Ab initio molecular dynamics (AIMD) as implemented in Quantum-ESPRESSO (QE) code [1] with the projector augmented-wave (PAW) method [2,3]. The exchange-correlation "XC" functional in the form of vdW-optB86b was used for all DFT simulations in this study [2–5]. A kinetic-energy cutoff of 50 Ry was selected for the plane-wave basis set. We used a unit cell in all our calculations. The initial atomic positions and cell sizes of the investigated unit cell were determined from the geometry optimization calculations by DFT. AIMD simulations were performed within the CP molecular dynamics code [1] as implemented in the QE package. All AIMD simulations performed with canonical ensemble (NVT) and temperature was controlled at 300 K. Larger time steps are needed for the AIMD computations to obtain reliable thermal stability. So, the run time is set at 500 ps. Thermal stability was estimated using the linear part of slopes of total energy and temperature where the system reaches equilibrium.

The formation process of ABX3 perovskite structures:

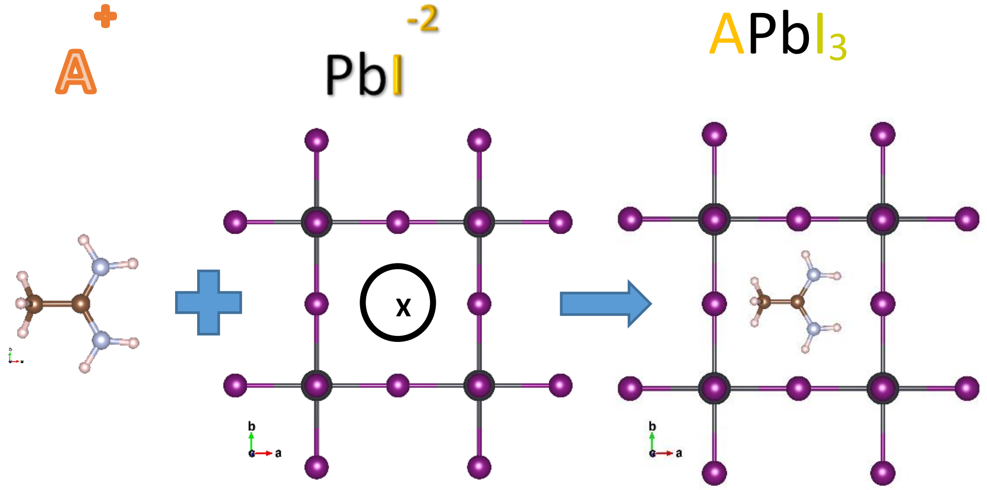

Figure S1: The process of merging a cation A^+ into the PbI_6^- framework to produce perovskite structures.

The formation process of all the studied structures was performed by replacing the "A+" cation in the "PbI6-" framework, which results in the full shape as illustrated in Figure S1 [6,7].

Tolerance factor (Goldschmidt factor) and octahedral factor calculations method:

In an ideal perovskite of ABX_3 composition, the radii of all the cations and anions should match the tolerance factor t expressed as:

$$t = \frac{(r_A + r_X)}{\sqrt{2}(r_B + r_X)}, \qquad (S1)$$

and octahedral factor μ which can be obtained by:

$$\mu = \frac{r_B}{r_X}, \qquad (S2)$$

where the ionic radii of A, B, and X are denoted by r_A, r_B, and r_X, respectively [8,9].

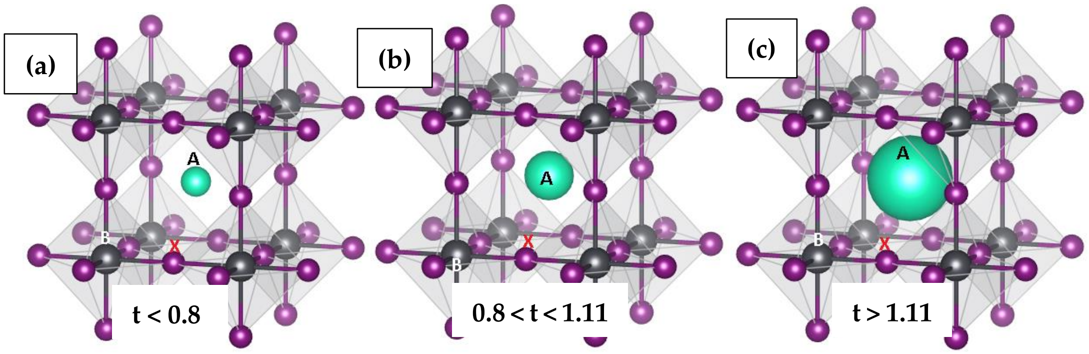

Figure S2: Ionic packing in an ideal cubic perovskite structure, with (a) a loosely packed crystal structure with a small A radius with $t < 1$, (b) an ideal cubic perovskite structure with $0.8 < t < 1.11$ and (c) a tightly packed crystal structure with a large A radius with $t > 1$.

Optical calculations method:

Optical constants can be calculated from the dielectric function. The energy loss spectrum *L(ω)*, reflectance *R(ω)*, refractive index *n(ω)*, and absorption coefficient *α(ω)* can be derived using the $\varepsilon_1(\omega)$ and $\varepsilon_2(\omega)$ parameters by the formulas:

$$\alpha(\omega) = \frac{\sqrt{2}\omega}{c}\left\{\left[\varepsilon_1^2(\omega) + \varepsilon_2^2(\omega)\right]^{\frac{1}{2}} - \varepsilon_1(\omega)\right\}^{\frac{1}{2}}, \qquad \text{(S3)}$$

$$\alpha(\omega) = \frac{\sqrt{2}}{c}\left\{\left[\varepsilon_1^2(\omega) + \varepsilon_2^2(\omega)\right]^{\frac{1}{2}} - \varepsilon_1(\omega)\right\}^{\frac{1}{2}}, \qquad \text{(S4)}$$

$$R(\omega) = \left|\frac{\sqrt{\varepsilon_1(\omega) + i\varepsilon_2(\omega)} - 1}{\sqrt{\varepsilon_1(\omega) + i\varepsilon_2(\omega)} + 1}\right|^{\frac{1}{2}}, \qquad \text{(S5)}$$

$$L(\omega) = \frac{\varepsilon_2(\omega)}{\varepsilon_1^2(\omega) + \varepsilon_2^2(\omega)}, \qquad \text{(S6)}$$

Power conversion efficiency calculations method:

J_{SC} can also be obtained using Eq. 7:

$$J_{SC} = e \times (F_{cell} - F_0), \qquad \text{(S7)}$$

where F_{cell} is photon flux emitted by the solar and F_0 is the photon flux at zero applied voltage.

The short-circuit current J_{SC} can be then expressed as:

$$J_{SC} = \frac{2\pi f e}{c^2 h^3}\left(1 - e^{-\alpha(\epsilon)L}\right) \times \left(\int_{E_g}^{\infty} \frac{\epsilon^2}{\left(\left(e^{\left(\frac{\epsilon}{k_B T_S}\right)} - 1\right)\right)} d\epsilon - n_r^2(\epsilon)\int_{E_g}^{\infty} \frac{\epsilon^2}{\left(\left(e^{\left(\frac{\epsilon}{k_B T}\right)} - 1\right)\right)} d\epsilon\right), \qquad \text{(S8)}$$

The open-circuit voltage V_{OC} according to the Shockley-Queisser limit can be calculated by Eq. 9

$$V_{OC} = \frac{KT_{cell}}{e} ln\left(\frac{F_{cell}}{F_0}\right), \qquad \text{(S9)}$$

The fill factor of the solar cell is given by Eq. 10:

$$FF = \frac{max[j(V) \times V]}{J_{SC} \times V_{OC}}, \qquad \text{(S10)}$$

The power conversion efficiency of the solar can be calculated by Eq. 11:

$$\eta = \frac{max[j(V) \times V]}{P_S}, \qquad \text{(S11)}$$

where e is the electron charge, E is incident photon energy, and P_S the power that the sun radiates on Earth per unit area, respectively [10–12]:

$$P_S = \frac{2\pi f}{c^2 h^3}\int_{E_g}^{\infty} d\epsilon \frac{\epsilon^2}{\left(e^{\left(\frac{\epsilon}{k_B T_S}\right)} - 1\right)}, \qquad \text{(S12)}$$

Difference charge calculations method:

The yellow region represents charge accumulation, whereas the blue region represents charge depletion. Charge redistribution occurred mostly between the A+ cation and the PbI_6- anion. The change

of charge density between the A+ cation and the PbI_6- anion reveals that electrons are mostly transferred from the blue range (cation) to the yellow range (anion) as demonstrated in Figure S3.

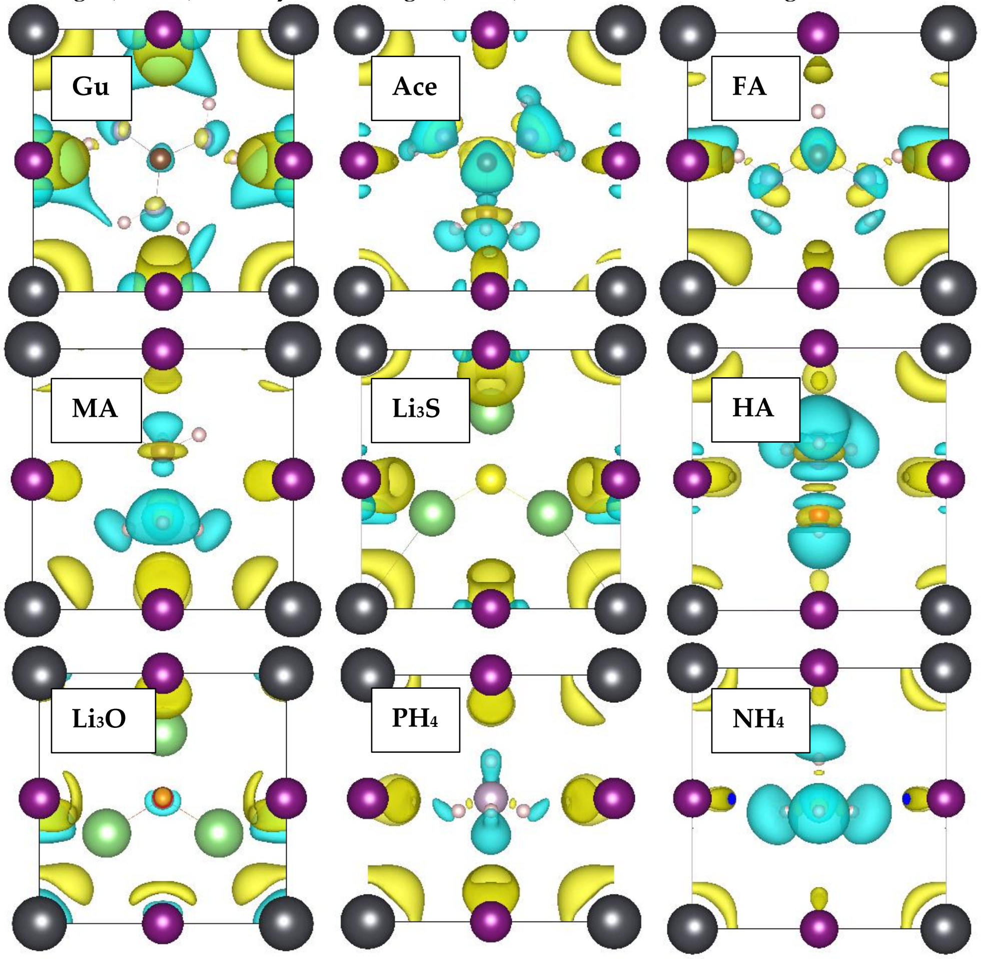

Figure S3 Difference in charge between the A+ cation and the PbI_6- anion, where the yellow area represents charge accumulation, whereas the blue area represents charge depletion.

1. Giannozzi, P.; Baroni, S.; Bonini, N.; Calandra, M.; Car, R.; Cavazzoni, C.; Ceresoli, D.; Chiarotti, G.L.; Cococcioni, M.; Dabo, I.; et al. QUANTUM ESPRESSO: A Modular and Open-Source Software Project for Quantum Simulations of Materials. *Journal of Physics: Condensed Matter* **2009**, *21*, 395502, doi:10.1088/0953-8984/21/39/395502.
2. Blöchl, P.E. Projector Augmented-Wave Method. *Physical Review B* **1994**, *50*, 17953–17979, doi:10.1103/PhysRevB.50.17953.
3. Perdew, J.P.; Burke, K.; Ernzerhof, M. Generalized Gradient Approximation Made Simple. *Physical Review Letters* **1996**, *77*, 3865–3868, doi:10.1103/PhysRevLett.77.3865.
4. Kresse, G.; Joubert, D. From Ultrasoft Pseudopotentials to the Projector Augmented-Wave Method. *Physical Review B* **1999**, *59*, 1758–1775, doi:10.1103/PhysRevB.59.1758.
5. Dion, M.; Rydberg, H.; Schröder, E.; Langreth, D.C.; Lundqvist, B.I. Van Der Waals Density Functional for General Geometries. *Physical Review Letters* **2004**, *92*, 246401, doi:10.1103/PhysRevLett.92.246401.
6. Singh, D.J.; Park, C.H. Polar Behavior in a Magnetic Perovskite from A-Site Size Disorder: A Density Functional Study. *Physical Review Letters* **2008**, *100*, 3–6, doi:10.1103/PhysRevLett.100.087601.
7. Peña, M.A.; Fierro, J.L.G. Chemical Structures and Performance of Perovskite Oxides. *Chemical Reviews* **2001**, *101*, 1981–2017, doi:10.1021/cr980129f.
8. Sani, F.; Shafie, S.; Lim, H.N.; Musa, A.O. Advancement on Lead-Free Organic-Inorganic Halide Perovskite Solar Cells: A Review. *Materials* **2018**, *11*, 1–17, doi:10.3390/ma11061008.
9. Wikipedia, F. Ionic Radius. *Wiki* **1000**, 1–10.
10. Pbi, N.H.; Weller, M.T.; Weber, O.J.; Frost, J.M.; Walsh, A. Cubic Perovskite Structure of Black Formamidinium Lead Iodide, α - [HC(NH 2) 2]PbI 3 , at 298 K. **2015**, 4–7, doi:10.1021/acs.jpclett.5b01432.
11. Zhou, Y.; Chen, J.; Bakr, O.M.; Sun, H.T. Metal-Doped Lead Halide Perovskites: Synthesis, Properties, and Optoelectronic Applications. *Chemistry of Materials* **2018**, *30*, 6589–6613, doi:10.1021/acs.chemmater.8b02989.
12. Peng, C.; Chen, J.; Wang, H.; Hu, P. First-Principles Insight into the Degradation Mechanism of $CH_3NH_3PbI_3$ Perovskite: Light-Induced Defect Formation and Water Dissociation. *The Journal of Physical Chemistry C* **2018**, acs.jpcc.8b07294, doi:10.1021/acs.jpcc.8b07294.
